# Wearable Sensors Improve Prediction of Post-Stroke Walking Function Following Inpatient Rehabilitation

**DOI:** 10.1109/JTEHM.2022.3208585

**Published:** 2022-09-22

**Authors:** Megan K. O’Brien, Sung Y. Shin, Rushmin Khazanchi, Michael Fanton, Richard L. Lieber, Roozbeh Ghaffari, John A. Rogers, Arun Jayaraman

**Affiliations:** Max Nader Laboratory for Rehabilitation Technologies and Outcomes ResearchShirley Ryan AbilityLab 505538 Chicago IL 60611 USA; Department of Physical Medicine and RehabilitationNorthwestern University Chicago IL 60611 USA; NOV, Inc. Houston TX 77064 USA; Feinberg School of MedicineNorthwestern University Chicago IL 60611 USA; Alife Health San Francisco CA 94123 USA; Department of Biomedical EngineeringNorthwestern University Evanston IL 60208 USA; Querrey Simpson Institute for Bioelectronics, Northwestern University Evanston IL 60208 USA; Department of Materials Science and EngineeringNorthwestern University Evanston IL 60208 USA; Department of ChemistryNorthwestern University Evanston IL 60208 USA; Department of Mechanical EngineeringNorthwestern University Evanston IL 60208 USA; Department of Electrical Engineering and Computer ScienceNorthwestern University Evanston IL 60208 USA

**Keywords:** Accelerometers, digital health, machine learning, stroke (medical condition), wearable sensors

## Abstract

Objective: A primary goal of acute stroke rehabilitation is to maximize functional recovery and help patients reintegrate safely in the home and community. However, not all patients have the same potential for recovery, making it difficult to set realistic therapy goals and to anticipate future needs for short- or long-term care. The objective of this study was to test the value of high-resolution data from wireless, wearable motion sensors to predict post-stroke ambulation function following inpatient stroke rehabilitation. Method: Supervised machine learning algorithms were trained to classify patients as either household or community ambulators at discharge based on information collected upon admission to the inpatient facility (N=33-35). Inertial measurement unit (IMU) sensor data recorded from the ankles and the pelvis during a brief walking bout at admission (10 meters, or 60 seconds walking) improved the prediction of discharge ambulation ability over a traditional prediction model based on patient demographics, clinical information, and performance on standardized clinical assessments. Results: Models incorporating IMU data were more sensitive to patients who changed ambulation category, improving the recall of community ambulators at discharge from 85% to 89-93%. Conclusions: This approach demonstrates significant potential for the early prediction of post-rehabilitation walking outcomes in patients with stroke using small amounts of data from three wearable motion sensors. Clinical Impact: Accurately predicting a patient’s functional recovery early in the rehabilitation process would transform our ability to design personalized care strategies in the clinic and beyond. This work contributes to the development of low-cost, clinically-implementable prognostic tools for data-driven stroke treatment.

## I. Introduction

Inpatient stroke rehabilitation is an early-stage program integrating clinical care with targeted therapy to maximize a patient’s functional recovery. A primary goal of inpatient rehabilitation is to retrain patients to maneuver safely and independently in the home and community after hospital discharge, such as by restoring walking ability. Walking at home or in the community are very meaningful tasks for individuals following a stroke; however, they are uniquely different skills. The functional capacity, coordination, endurance, strength, motor control, cognition needed are significantly higher for community ambulation compared to household ambulation [1], [2], [3]. With the average length of stay at an inpatient rehabilitation facility (IRF) in the United States declining over time [4], [5] (current estimates ranging from 8.9-22.2 days depending on impairment severity [6]), the patient’s care team only have a brief time to create and execute a support plan for discharge based on the patient’s capability. For patients who do not achieve functional independence, including community walking, comprehensive discharge plans must be established to ensure appropriate support or continued services outside of the hospital. Accurately predicting a patient’s outcomes and response to treatment early in the rehabilitation process would be invaluable for setting realistic and achievable goals during therapy, anticipating future needs for assistive equipment (e.g., wheelchair, walker, orthotics) or home modifications, maximizing time for caregiver training, and informing interactions with insurance providers.

**FIGURE 1.**
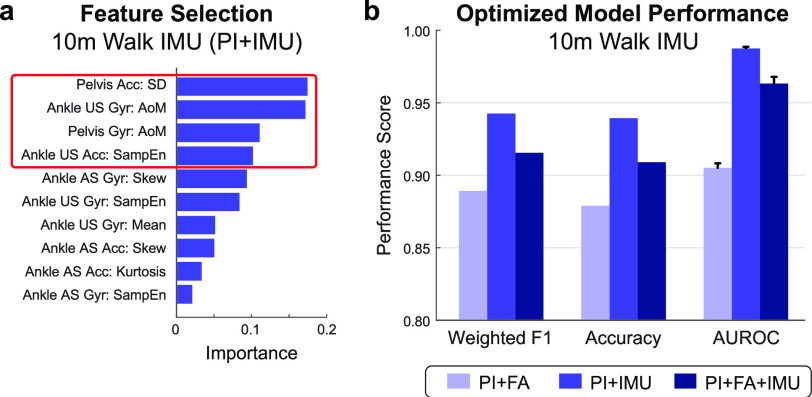
**Discharge predictions using fixed distance IMU data**. **(a)** Top 10 important features for a model utilizing patient information (PI) and IMU data. Features from IMUs placed on the pelvis and unaffected ankle were selected via backward elimination for optimized model performance (red box). **(b)** Models trained with IMU sensor data from 10 m of walking at admission show improved classification of discharge walking level over a model using patient information and functional assessment scores (FA) alone. Bars show the average and SD of each metric across 100 iterations. *Acc* = Accelerometer; *Gyr* = Gyroscope; *US* = Unaffected Side; *AS* = Affected Side; *AoM* = Amount of motion; *SampEn* = Sample entropy.

Numerous prediction models have been proposed in stroke research. Many of these models are based exclusively on information readily available from an electronic medical record (EMR), including patient demographics, stroke characteristics, and standardized functional assessment scores [7], [8]. For instance, Bland *et al.* identified that a Berg Balance Scale (BBS) score ≤20 points and a Functional Independence Measure (FIM) walk item score of 1 or 2 at IRF admission were predictive of low functional ambulation at discharge [9]. Harari *et al.* found that functional assessment scores recorded at admission to an inpatient rehabilitation facility (IRF) were the most important predictors of the same test scores at discharge, over age, stroke characteristics, or performance on other assessments of gait or balance [10]. While standardized clinical functional assessments are useful indicators to predict future outcomes, their administration can be time-intensive and cumbersome due to limited interaction time with patients and need for specialized training. Furthermore, some functional assessments are scored using subjective rating scales and suffer from floor/ceiling effects [11], high inter-/intra-rater variability [12], [13], and lack of suitability for patients with severe cognitive impairments [14]. Reliance on these assessments could result in imprecise and inequitable prognoses. Indeed, previous studies indicate that patient prognosis can be an important source of variation in healthcare and may lead to inconsistent access to rehabilitation services across the continuum of care [15], [16]. Identifying alternative, objective predictors of stroke recovery that can be obtained easily in a clinical setting may improve measurement resolution, diagnostic accuracy, and lead to data-driven, prognostic models.

Wearable sensors have started transforming our ability to objectively measure patient health and performance in clinical settings. Ongoing technological advances yield sensors that are smaller and more affordable, with options to wirelessly stream analytics to customized, user-friendly digital dashboards. Inertial measurement units (IMUs) are especially ubiquitous in research-grade and commercial devices, providing three-dimensional kinematic metrics from accelerometers and gyroscopes. These devices have demonstrated utility in various stroke rehabilitation applications in the inpatient setting – for example: instrumenting clinical tests [17]; measuring changes in motor features related to arm reaching [18], gait and transfers [19], [20], or balance [21]; and detecting posture or activity [22]. To date, there has been limited exploration of IMU data for recovery prediction after stroke. A recent study in exception found that combining clinical data obtained at admission with inertial sensor data provided superior prediction of the discharge FIM compared to clinical data alone [23], indicating that wearable sensors could be beneficial for prognostic models. It remains to be seen whether IMU data can characterize recovery of walking ability, a critical discharge need for individuals following a stroke. High resolution measures of lower-limb motion may capture nuanced information about a patient’s propensity for functional gait recovery.

Thus, the objective of this study was to quantify the value of inertial sensor data in predicting post-stroke recovery of walking function. We trained machine learning models to retrospectively classify functional walking ability at IRF discharge (household or community ambulation) using various types of data obtained at admission, including patient demographics and clinical information, functional assessment scores, and IMU sensor data. We hypothesized that IMU data recorded during a brief walking bout at IRF admission would improve discharge predictions over traditional models trained using standard clinical assessment scores and patient characteristics alone.

## II. Results

### A. IMU Data Recorded During 10-Meter Walk at Admission Improve Predictions of Functional Walking Level at Discharge

We trained a machine learning algorithm to classify patients as household ambulators (discharge 10MWT score ≤0.4 m/s) or community ambulators (discharge 10MWT > 0.4 m/s) [1], [24] at discharge using input data recorded at admission.

A balanced random forest classifier was selected as the algorithm of choice following initial exploration (see *V. Methods and Procedures*, section *F. Algorithm Selection*), demonstrating the highest average weighted F1 score and performance stability. Features for model training included patient information (PI), functional assessment scores at admission (FA), and metrics computed from inertial sensor data during the 10MWT at admission (IMU, recorded from the pelvis and bilateral ankles). Thus, IMU features characterized lower limb walking motion at admission over a fixed 10-m distance.

Model performance was evaluated using the weighted F1 score, accuracy, and area under the receiver operating characteristic (AUROC). All metrics were computed using leave-one-subject-out cross-validation ( $\text{N}=33$ patients) following optimization via feature selection and hyperparameter tuning. These procedures were repeated over 100 iterations with incremented random seeds to account for the stochasticity of the balanced random forest classifier, and model performance metrics were averaged.

**FIGURE 2.**
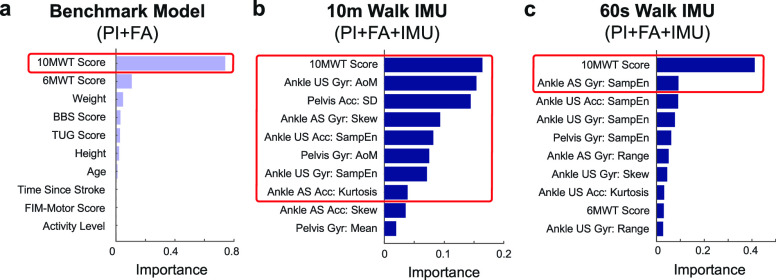
**Feature importance and selected features for the benchmark and full model**. Red box indicates the features selected via backward elimination for use in the optimized model training and testing. **(a)** Benchmark model (PI+FA), with 1 feature selected (10MWT score at admission), **(b)** Fixed distance full model (PI+FA+IMU), with 8 features selected (10MWT score at admission and 7 features from the ankles and pelvis IMUs), **(c)** Fixed duration full model (PI+FA+IMU), with 2 features selected (10MWT score at admission and a feature from the affected ankle IMU). No patient information (PI) features were selected for use in any model. *Acc* = Accelerometer; *Gyr* = Gyroscope; *US* = Unaffected Side; *AS* = Affected Side; *AoM* = Amount of motion; *SampEn* = Sample entropy.

Example feature importance and selection are illustrated in **Fig. 1a** for a model trained on patient information and IMU features (PI+IMU). Four features were selected for this model via backward elimination, including the standard deviation of acceleration at the pelvis, amount of motion of the stroke-unaffected ankle, amount of motion of the pelvis, and sample entropy of acceleration on the stroke-unaffected ankle. Feature importance and selection for the PI+FA model and PI+FA+IMU fixed distance model are provided in **Fig. 2a** and **Fig. 2b**, respectively.

**Fig. 1b** compares optimized model performance across three different combinations of feature training sets. Performance was highly stable across the 100 iterations for all models, with no fluctuation in the participant classifications. The benchmark model, trained on patient information and functional assessment scores only (PI+FA), had a weighted F1 score of 0.889. Adding sensor-based features – either to the patient information alone (PI+IMU) or to both patient information and functional assessment scores (PI+FA+IMU) – improved the weighted F1 to 0.943 and 0.916, respectively. This trend was preserved for other metrics of model performance, including accuracy (PI+FA: 0.879; PI+IMU: 0.939; PI+FA+IMU: 0.909) and AUROC (PI+FA: 0.905± 0.003; PI+IMU: 0.988± 0.001; PI+FA+IMU: 0.963± 0.005).

### B. Models Trained on IMU Data Were More Accurate in Classifying Patients Who Improved Functional Walking Level During Rehabilitation

**FIGURE 3.**
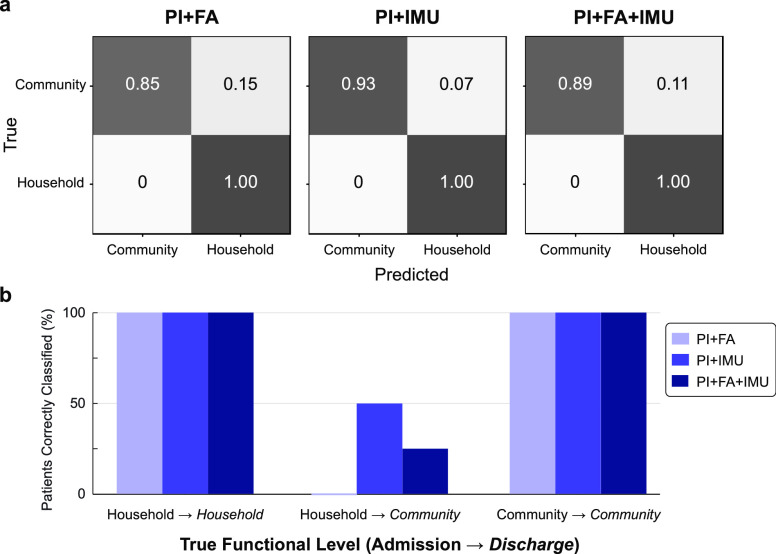
**Model performance for household and community ambulators**. **(a)** Confusion matrices for each model. **(b)** Percentage of patients correctly classified based on functional walking categories at IRF admission and discharge. While all models correctly classified 100% of patients who maintained the same level of walking function (Household $\to $ *Household*, $\text{N}=8$; Community $\to $ *Community*, $\text{N}=21$), only models that included IMU data were able to identify any patients who changed functional walking level during IRF treatment (Household $\to $ *Community*, $\text{N}=4$).

The benchmark PI+FA model correctly predicted the discharge functional walking level for 23 out of 27 community ambulators (85%) and for 6 out of 6 household ambulators (100%) (**Fig. 3a**). Adding sensor data improved the recall of discharge community ambulators to 25 out of 27 (93%) for PI+IMU or 24 out of 27 (89%) for PI+FA+IMU without compromising the perfect recall of household ambulators.

**FIGURE 4.**
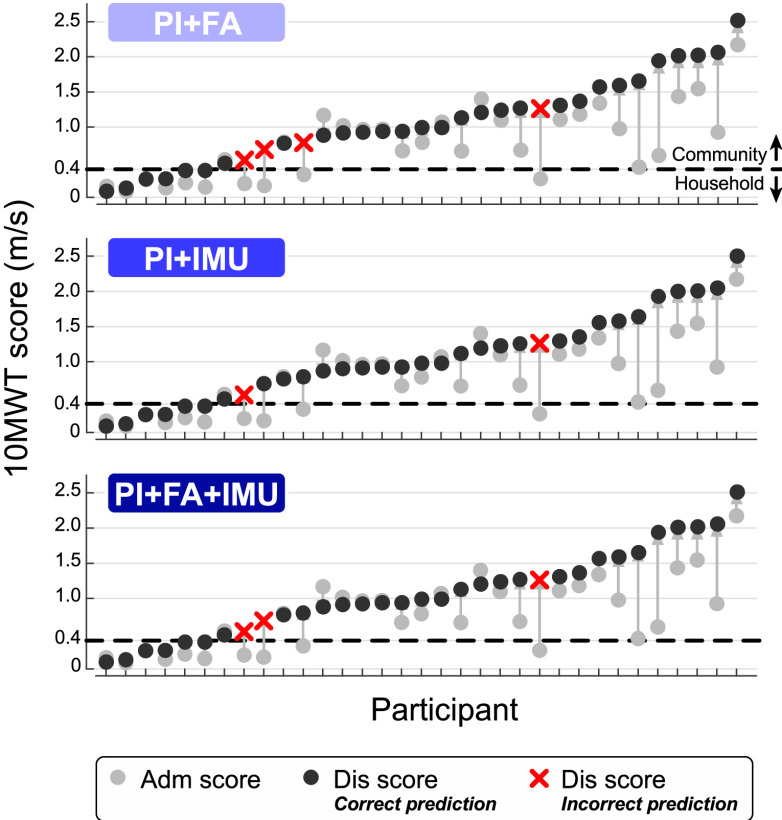
**Model predictions by participant**. Correct and incorrect predictions for each participant’s discharge ambulation ability in relation to their Adm and Dis 10WMT scores (fixed distance model). Dashed lines illustrate the 0.4 m/s threshold that differentiates the community and household ambulator classes.

We next examined the models’ ability to detect *changes* in walking function during IRF treatment. All models achieved perfect recall for patients who maintained the same level of walking function between admission and discharge, which applied to most study participants (community: $\text{N}=21$; household: $\text{N}=8$). However, only models that included IMU data could correctly classify patients who changed functional walking level between admission and discharge (progressed from household to community ambulators: $\text{N}=4$). The PI+IMU model correctly classified two out of these four patients (50%), while the PI+FA+IMU model correctly classified 1 out of 4 (25%). The benchmark PI+FA model was unable to correctly classify any of these patients, instead predicting that they would remain at the household functional level (**Fig. 3b**). Such misclassifications were exclusively tied to the four patients who transitioned to a higher level of functional ambulation. These patients exhibited a moderate range of 10MWT scores at Adm and Dis relative to the 0.4 m/s classification threshold (**Fig. 4**). The two patients consistently misclassified across all models had Dis scores close to (0.54 m/s) and far from (1.27 m/s) the threshold, with similar scores at Adm (0.20 and 0.26 m/s, respectively). Adding IMU features to the model reduced misclassification for the other two patients with Adm scores close to (0.33 m/s) and far from (0.17 m/s) the threshold, with intermediate Dis scores (0.79 and 0.69 m/s, respectively).

### C. Longer Bouts of Walking at IRF Admission Did Not Improve Gait Speed Classifications

**FIGURE 5.**
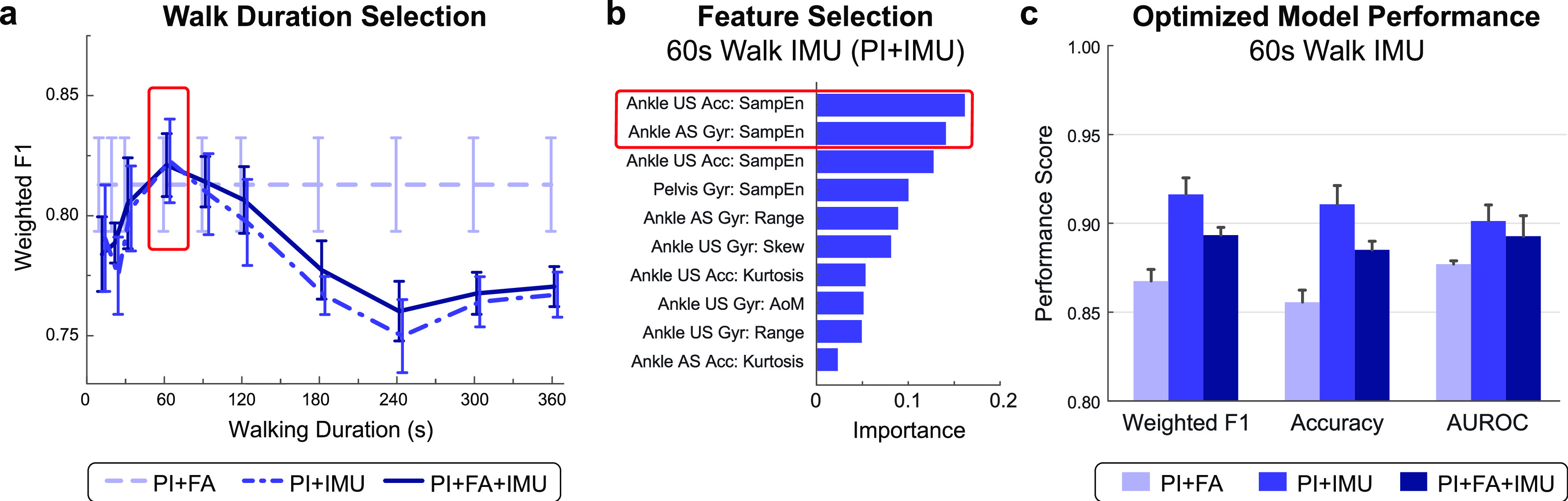
**Discharge prediction performance using fixed duration IMU data**. **(a)** Maximum model performance was observed using the first 60 s of IMU data (red box) recorded during a 6MWT at IRF admission. **(b)** Top 10 important features for the PI+IMU model using 60 s of IMU walking data. Features from the bilateral ankles were selected via backward elimination for optimized model performance (red box). **(c)** Models trained with 60 s of IMU walking data also show improved prediction of discharge walking level over the benchmark PI+FA model. Bars show the average and SD of each metric across 100 iterations. Note that the PI+FA fixed duration model performance (shown here) varies slightly from the fixed distance model (Fig 1b) since data from a larger number of patients were available for training.

To examine the impact of amount of sensor data on model performance, we also utilized IMU data recorded during different walking durations ranging from 10–360 s, obtained from a 6MWT. Data from two additional patients were available for this fixed duration paradigm, so all models were trained, optimized, and tested using the available data from a larger patient cohort ( $\text{N}=35$). Pre-optimized model performance is shown in **Fig. 5a** for each model and walking duration. The 60-s walking duration was selected for downstream analysis since this duration exhibited the highest initial classification performance.

Example feature importance and selection is illustrated in **Fig. 5b** for the fixed duration model trained on patient information and IMU features computed from 60 s of walking (PI+IMU). Two features were selected for this model via backward elimination, including the sample entropy of acceleration on the stroke-unaffected ankle and sample entropy of rotational velocity on the stroke-affected ankle. Feature importance and selection for the PI+FA model and PI+FA+IMU fixed duration model are provided in **Fig. 2a** and **Fig. 2c**, respectively.

Similar to the fixed distance (10-m walk) analysis, the fixed duration (60-s walk) analysis revealed improved classification for models incorporating sensor data compared to the benchmark PI+FA model (**Fig. 5c**). Optimized models with IMU features demonstrated higher weighted F1 score (PI+FA: 0.867± 0.007; PI+IMU: 0.916± 0.009; PI+FA+IMU: 0.893± 0.004), accuracy (PI+FA: 0.855± 0.007; PI+IMU: 0.911± 0.011; PI+FA+IMU: 0.885± 0.005) and AUROC (PI+FA: 0.877 ± 0.002; PI+IMU: 0.901± 0.009; PI+FA+IMU: 0.893± 0.012). The fixed duration models did not outperform the fixed distance models (**Fig. 1b**).

## III. Discussion

We found that inertial sensor data recorded from the bilateral ankles and pelvis during a brief walking bout at IRF admission improved the prediction of discharge walking ability. Specifically, models trained with sensor data (PI+FA+IMU, PI+IMU) were better able to predict household or community ambulation at discharge compared to a model relying on patient information and admission functional assessment scores alone (PI+FA). This trend was true whether IMU data were recorded over a fixed walking distance (10 m) or a fixed walking duration (60 s). Improved model performance with IMU data stemmed from superior identification of patients who improved functional walking level during inpatient rehabilitation (progressing from household ambulation at admission to community ambulation at discharge).

The best-performing model utilized patient information and IMU data recorded during a 10 m walk (fixed distance PI+IMU), indicating that functional assessment scores may not be necessary for accurate predictions relating to walking function. Sensor data recorded during a fixed duration of walking also improved prediction performance over the benchmark PI+FA model, though not beyond the fixed distance walking data. A model utilizing 60 s of walking was optimal for this approach, with longer walking bouts reducing performance below the benchmark. These results are relatively intuitive since the model is trained to predict gait speed from a 10MWT, whereas longer bouts of walking rely more strongly on patient endurance. While performance on gait speed and endurance tests are correlated [25], [26], it is likely that fatigue and fluctuations in gait speed during the 6MWT negatively affected model performance for longer bouts of walking. Because patients walk at different self-selected gait speeds, the fixed duration analysis captures different amounts of walking within the same amount of time. Future work may also consider IMU data from alternative walking strategies, such as recording the same number of steps between patients.

Interestingly, we observed worse performance in the PI+FA+IMU model compared to the PI+IMU model. This drop in performance is attributed to the PI+FA+IMU model misclassifying a participant who transitioned to the community ambulation class (10MWT score increasing from 0.17 m/s at admission to 0.69 m/s at discharge, as shown in **Fig. 4**). A possible explanation is that adding the admission 10MWT score to IMU features biased the model toward a household ambulation prediction for this participant, since they had relatively low score at admission. However, a larger sample of patients who transition ambulation categories is needed to test this possibility and refine the models. Our quantized feature selection method may also contribute to the discrepancy between these two models. Future work will explore other forms of regularization in the models to assess the consistency of this behavior.

An estimated 70-80% of patients are able to walk at the chronic stage of stroke [27]; however, only 30-50% of patients recover community walking function [1], [28]. A crucial test of a model’s predictive power is whether it can correctly identify patients who change categories, such as from household to community ambulators. Previous work showed that functional ambulation is correlated with overall mobility and quality of life, especially for household ambulators transitioning to higher ambulation categories [29]. Predicting early in the acute rehabilitation program whether a participant will achieve community-level ambulation or remain a household ambulator at IRF discharge would help clinicians develop targeted treatment and care planning strategies for patients and their families.

For models utilizing IMU data, sample entropy and amount of motion features were consistently ranked among the most important. Higher sample entropy (greater complexity in the movements) and greater overall motion were associated with the higher ambulation level. Additional data and feature exploration will be critical to establish the most important features across a larger sample size. To reduce the feature space and risk of overfitting, we computed features using the magnitude of the acceleration and gyroscope signals, rather than on the three sensor axes. Future work examining motion in different anatomical planes (i.e., anteroposterior, mediolateral, and vertical movements of the pelvis and ankles) will be of interest to illuminate additional predictors of recovery based on detailed gait patterns. As expected, for any models utilizing the functional assessment scores, we found that the strongest predictor of functional walking level at discharge – which was defined using the discharge 10MWT score – was the 10MWT score at admission. This aligns with our previous work, which demonstrated that a functional assessment score at admission was the strongest predictor of a patient’s performance on that same assessment at discharge, over other functional assessments and patient information such as demographics, stroke presentation, and pre-morbid activity levels [10]. Patient information, such as age, height, or stroke characteristics, were not selected as important features for any model, suggesting that this information is less predictive than measures of gait function and behavior. Indeed, a model trained on PI alone demonstrated substantially lower precision and recall than models including FA and IMU data in the fixed distance model (see *V. Methods and Procedures*, section *F. Algorithm Selection*).

Recently, quantitative measures of kinematic, biomechanical, or neurological factors have also demonstrated sensitivity in predicting post-stroke recovery outcomes. These models incorporate data from force plates [30], 3D motion tracking [31], [32], or brain stimulation technology [33]. However, the high cost, low portability, and technical demands of these in-depth measurement systems can make them impractical to implement in a typical rehabilitation facility. Unobtrusive, affordable wearable sensors with automated data streaming may be a more practical alternative to capture early biomarkers of impairment and recovery during simple activities that are regularly performed in the rehabilitation program.

The primary limitation of this study is the small sample size of patients, which could contribute to overfitting and limit our ability to make claims about the most valuable predictive sensor features for a robust patient population. With a small sample size and imbalanced class distribution, the random seeding of the explored machine learning algorithms had an observable impact on classification performance. Rather than setting the seed to a single value, we iterated the models over 100 different random seeds and computed average performance to mitigate the effects of model stochasticity. This leads to our second limitation, which is that models were optimized using data from all participants to select features and hyperparameters, resulting in some data leakage between the training and test sets. We selected this approach over nested optimization to create single, aggregate models for easy interpretation and comparison. We did not use a hold-out set to maximize the data available for algorithm training with this small sample size. Although we do not expect the resulting data leakage to impact our broader findings that IMU data improved predictions, subsequent analyses will aim to increase the sample size and explore other modeling strategies to avoid these issues. Finally, it should be noted that all models perform relatively well for this simple classification problem. Indeed, our ability to compare the models hinges on their predictions for a small sample of patients – namely, the four patients who transition ambulation categories (household to community) between IRF admission to discharge.

In the present study, we excluded data from patients who were unable to complete the either the 10MWT or 6MWT, utilizing IMU data during these walking assessments to train and test the predictive models. As such, these models require patients to be ambulatory at IRF admission, which is not always the case. For example, in a study of 41 IRFs, approximately 6% of stroke survivors were unable to ambulate or required assistance at admission [34]. It remains to be seen whether wearable sensor data have predictive value for non-ambulatory patients; incorporating sensor data during alternative activities, such as sitting [35], [36], may facilitate the prediction of walking recovery for these patients.

Importantly, this model was trained using admission and discharge data from patients at a single rehabilitation hospital, which may limit its generalizability to broader post-stroke outcomes. Stinear *et al.* [8] note the importance of predicting outcomes at specific time-points after stroke rather than at discharge, since discharge itself is linked to functional achievements and subject to variations in care structure and resources. We have developed a model that intentionally leverages predictions based on standard-of-care treatment at a single rehabilitation hospital. While it remains to be seen whether such a model will generalize to other IRFs, the approach described here can serve as a roadmap for the development of site-specific models for accurate, validated predictions at other rehabilitation hospitals.

Future work will expand the existing dataset for additional training and testing of the predictive model, including external validation in a new subset of patients. We will also test the predictive value of additional sensor data such as EMG or ECG to account for neuromuscular or cardiovascular factors, and we will examine the feasibility of regression models over classification models to improve the precision of predictions.

## IV. Conclusion

Inpatient stroke rehabilitation is often a hectic and overwhelming experience for patients, families, and clinicians working to deliver optimal therapeutic care. Many times, due to time restrictions, patients’ limited physical capabilities, or cognitive/communication impairments, full functional assessments and clinical measures are not recorded and/or uploaded to the EMR. Furthermore, the full sequence of assessments at admission might take as long as 2–3 hours to complete. This results in incomplete or inconsistent data, posing a significant challenge in the creation of traditional prediction models to estimate a patient’s future functional scores. Our current study suggests that a viable alternative is to record data from three simple inertial sensors during a brief walking bout (maximum of 60 seconds), which can be completed during any part of therapy or non-therapy time without significant dedicated time. This represents a unique translational engineering approach to support clinical evaluation and treatment of stroke using widely available IMU technology and machine learning techniques.

## V. Methods and Procedures

### A. Participants

Fifty-five patients with stroke at the Shirley Ryan AbilityLab (Chicago, IL, USA) enrolled in the study. Inclusion criteria were: having a diagnosis of stroke and undergoing acute inpatient rehabilitation at the Shirley Ryan AbilityLab; at least 18 years of age; and able and willing to give consent and follow study procedure directions. Exclusion criteria were: having a known neurodegenerative pathology (e.g., Alzheimer’s disease, Parkinson’s disease, etc.); pregnant or nursing; or utilizing a powered, implanted cardiac device for monitoring or supporting heart function. Medical clearance was obtained from each patient’s primary physician and all individuals (or a proxy) provided written informed consent prior to participation. The study was approved by the Institutional Review Board of Northwestern University (Chicago, IL; STU00205532) in accordance with federal regulations, university policies, and ethical standards regarding research on human subjects.

Data from 33 patients were available for the fixed distance analysis, after excluding patients who withdrew consent before the study (2 patients), were unable to complete a 10MWT at the admission time-point (13 patients), discharged without conducting discharge clinical tests (3 patients), and with incomplete sensor data (4 patients, e.g., due to depleted battery or sensor malfunction). Data from 35 patients were available for the fixed duration analysis, since two additional patients were able to perform a 6MWT at admission. This is because the 6MWT allows the patient to take rests as needed, whereas the 10MWT requires that the patient walk 10 m continuously, which fewer patients were able to complete at admission. Patient information (i.e., demographics, stroke characteristics) and functional assessment scores for the full cohort ( $\text{N}=35$) are provided in **Table 1**.

**TABLE 1:** Patient Information and Functional Assessment Scores at IRF Admission (N=35)

	Mean (SD)	Range
Age (years)	57.2 (13.9)	22–76
Sex (Female / Male)	16 / 19	N/A
Height (cm)	171.1 (11.4)	149.9–190.5
Weight (kg)	80.8 (19.5)	42.7–118
Length of IRF stay (days)	21.2 (8.8)	8–47
Time from stroke to admission (days)	9.5 (7.1)	3–35
Stroke type (Ischemic / Hemorrhagic / Both / Unclear)	28 / 4 / 1 / 2	N/A
Lesion location (Right / Left / Bilateral / Unknown)	18 / 12 / 3 / 2	N/A
Hemiparesis (Right / Left / Unknown)	10 / 15 / 10	N/A
Patient-reported premorbid lifestyle (Highly active / Moderately active / Sedentary)	12 / 7 / 16	N/A
10MWT^a^ (m/s)	0.73 (0.52)	0–2.2
6MWT $(m)$	162.5 (128.9)	10.5–461.7
FIM - Motor subscale (points)	42.5 (12.7)	20–73
BBS (points)	28.1 (15.0)	3-55
TUG^a^ (s)	30.75 (56.21)	0–314.57

$N/A =$ Not applicable; 10MWT = 10-Meter Walk Test; 6MWT = 6-Minute Walk Test; FIM = Functional Independence Measure; BBS = Berg Balance Scale; TUG = Timed Up and Go.

^a^ Patients unable to complete the assessment were assigned a score of 0.

### B. Data Collection and Experimental Setup

Within the first week of admission, patients performed a series of functional assessments for overground gait and balance, including the 10-Meter Walk Test (10MWT), 6-Minute Walk Test (6MWT), Berg Balance Scale (BBS), and Timed-Up-and-Go (TUG) in a non-standardized order based on the availability of equipment and space. The same clinical tests were administered within a week before discharge from the hospital to capture any functional changes following the inpatient rehabilitation process. We also collected the FIM motor subscore at admission and discharge from individual FIM items recorded in the patient’s EMR, in accordance with the Inpatient Rehabilitation Facility Patient Assessment Instrument guidelines (IRFPAI, regulated by the United States Centers for Medicare & Medicaid Services). All tests were administered and scored by a licensed physical therapist. Patient demographics and stroke information were obtained from the EMR and a study intake form.

**FIGURE 6.**
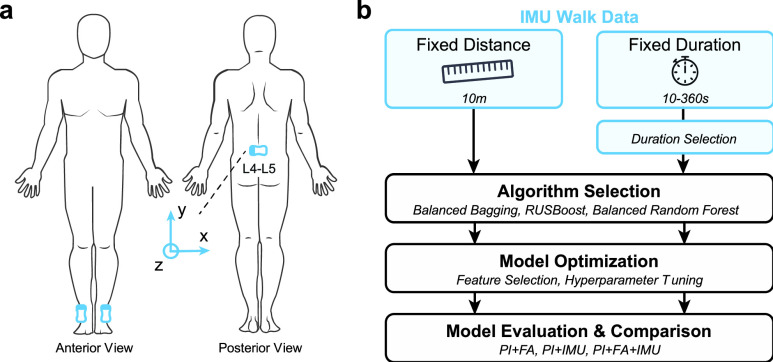
**Study overview**. **(a)** Placement of three wireless inertial measurement units (IMUs) at the pelvis and bilateral ankles. Coordinate system is shown for the pelvis sensor; ankle sensors are rotated by 90° clockwise. **(b)** Model pipeline for predicting discharge walking function and determining the relative value of IMU data. Separate models were trained using IMU data recorded during a fixed distance walk (10 m during the 10MWT) or a fixed duration walk (the first 10–360 s of the 6MWT) upon admission to a post-stroke IRF program.

During the clinical assessments at the admission, all participants wore three flexible, wireless inertial motion sensors (BioStampRC; MC10, Inc., Cambridge, MA) at the pelvis (L4-L5 region) and bilateral ankles (**Fig. 6a**). The sensors were attached to the skin with an adhesive film (Tegaderm; 3M, St. Paul, MN). The BioStampRC collected triaxial acceleration (sensitivity ±4g) and triaxial angular velocity (sensitivity ±2000°/s) at a sampling rate of 31.25 Hz. A Samsung tablet running the proprietary BioStampRC application was used to collect the sensor data and annotate the beginning and end of each trial or item of the clinical tests. De-identified sensor data were uploaded to the MC10 Cloud and then downloaded and stored on a HIPAA-compliant (Health Insurance Portability and Accountability Act of 1996) secure server.

### C. Feature Extraction

Three sets of features were defined and extracted from information obtained at admission, including patient information (PI, such as demographics and clinical information about their stroke), functional assessment scores (FA), and sensor data (IMU). **Table 2** summarizes the 71 total features utilized for model development. A custom code in MATLAB (Mathworks, Inc. R2017b, Natick, MA) calculated features from the sensor data and concatenated them with the other feature sets.

**TABLE 2:** Features Extracted for Prediction Models

Category	Mean (SD)	No. Features
Patient Information (PI)	Age, sex (female/male), height, weight, type of stroke (ischemic/hemorrhagic), time since stroke to admission, lesion location (right/left/bilateral/unknown), hemiparesis (right/left/unknown), patient-reported premorbid lifestyle (highly active/moderately active/sedentary)	9
Functional Assessments (FA)	Scores at admission: 10MWT, 6MWT, FIM (Motor subscale), BBS, TUG	5
Sensor data – Inertial Measurement Unit (IMU)	Acc and Gyr magnitude at admission walk test (fixed distance/duration): Mean, range, root-mean-square, standard deviation, skewness, kurtosis, sample entropy, dominant frequency and magnitude at (1) pelvis, (2) left ankle, (3) right ankle Gyr magnitude at admission walk test (fixed distance/duration): Amount of motion at (1) pelvis, (2) left ankle, (3) right ankle	57

10MWT = 10-Meter Walk Test; 6MWT = 6-Minute Walk Test; FIM = Functional Independence Measure; BBS = Berg Balance Scale; TUG = Timed Up and Go; Acc = Acceleration; Gyr = Gyroscope.

All sensor features were computed from the data recorded during the 10MWT (fixed walking distance) or a subset of the 6MWT (fixed walking duration). Sensor features included amount of motion (AoM) [37], defined as the cumulative angular displacement measured from gyroscope signals, and general statistical and mathematical features calculated from the gyroscope (Gyr) and accelerometer (Acc) signals of the IMU. All sensor features were computed from the magnitude of tri-axial signals to conserve the number of features and facilitate the compatibility of our analysis with alternative devices that may have different orientations or coordinate systems.

The analysis process is summarized in **Fig. 6b**. To evaluate the predictive value of sensor data in predicting discharge ambulation ability, we compared models with different sets of features, including: (1) patient information and functional assessments (PI+FA), (2) patient information and sensor data (PI+IMU), and (3) patient information, functional assessments, and sensor data combined (PI+FA+IMU). The PI+FA model served as a benchmark against which the other models, trained using sensor data, were compared.

An additional model, utilizing patient information only (PI), was also implemented for the fixed distance analysis. However, this model was not pursued further as it demonstrated low classification performance relative to the three models described above.

### D. Fixed Distance and Fixed Duration Analyses to Explore Alternative IMU Data Inputs

The 6MWT is a performance-based test of self-paced walking endurance, wherein the patient attempts to walk as far as they can in six minutes. Recording IMU data from a 6MWT provided a natural experiment to explore different durations of overground walking IMU data for use in the predictive models, as an alternative to a fixed distance walking bout afforded by the 10MWT.

Data from the full 6MWT were segmented into 10 different walking durations – 10, 20, 30, 60, 90, 120, 180, 240, 300, and 360 seconds from the beginning of the test – and IMU features were computed from the entire duration. Weighted F1 scores were computed across durations to select the best-performing algorithm and duration of walking data that would maximize model performance. Model optimization, evaluation, and comparison were then completed using IMU features computed from the selected duration.

### E. Classification Strategy

Walking speed is an objective indicator of post-stroke walking ability, a reliable marker of deficit severity, and a strong predictor of functional community ambulation [26], [29]. We targeted the classification of patients as household or community ambulators based on discharge 10MWT scores. Target model predictions were “household” or “community” discharge walking speed based on stratified 10MWT scores, in alignment with previous classifications for household ( $ < 0.4\text{m}$/s) and community (≥0.4 m/s) ambulation [1], [24].

For the fixed distance dataset, 26 participants were labeled as community walkers at discharge, and 7 participants were labeled as household walkers at discharge. For the fixed duration dataset, 28 participants were labeled as community walkers at discharge, and 7 participants were labeled as household walkers at discharge. These imbalanced classes can pose a challenge for machine learning models, with a risk of biasing classifications toward the majority class. To minimize this risk, we selected candidate algorithms that can contend with imbalanced classes, namely Balanced Random Forest, Balanced Bagging, and RUSBoost, which randomly undersamples from the majority class. All machine learning algorithms were implemented using the Scikit-Learn (0.23.2) and Imbalanced-Learn (0.23.2) libraries in Python (3.8.8).

### F. Algorithm Selection

**FIGURE 7.**
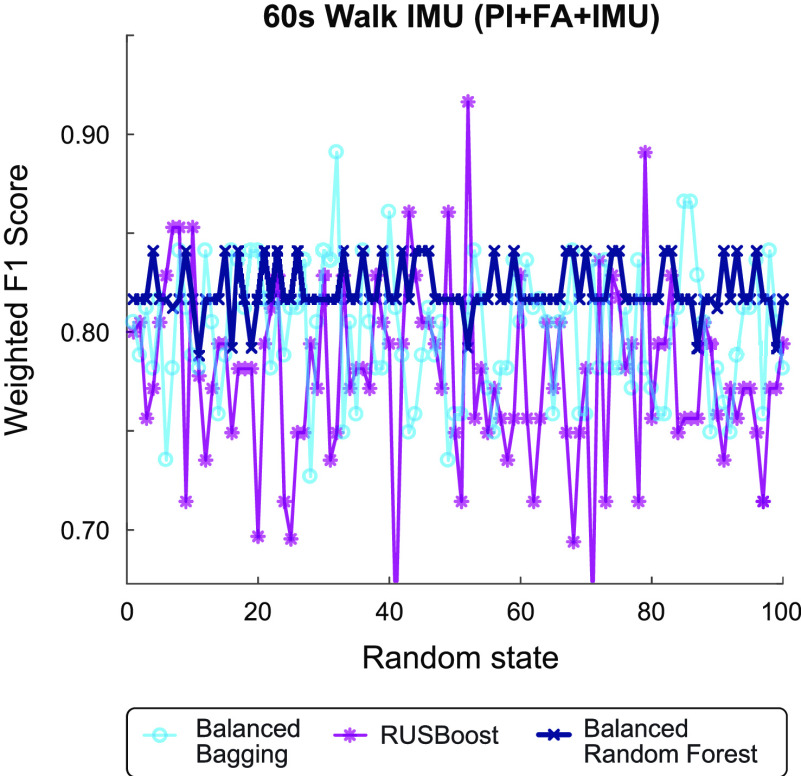
**Example effect of random seed state on model performance**. The stochastic nature of the explored algorithms causes variation in the weighted F1 score with random seeds. To evaluate broader performance of each model, performance was averaged across 100 repetitions with incrementing random seed. The Balanced Random Forest typically demonstrated higher average performance and lower fluctuation, as illustrated in this fixed duration model (60s walk) with all data inputs (PI+FA+IMU).

We evaluated the performance of each model using leave-one-subject-out cross validation. The primary performance metric was the weighted F1 score, an average of precision and recall scaled by the proportion of samples for each class. The weighted F1 score ranges from 0 to 1, with 1 indicating perfect precision and recall. Since the explored models are all stochastic in nature, the performance can vary depending on the random seed initialization, particularly for a small sample size with randomly sampled classes. For example, **Fig. 7** illustrates the variation in weighted F1 score across incrementing random seeds with PI+FA+IMU features (IMU features computed during a 60-s walk) applied on the three different algorithms. To cope with this issue and more broadly compare performance of each model, we executed 100 iterations with different random state parameters and computed the average and standard deviation in F1 score.

**FIGURE 8.**
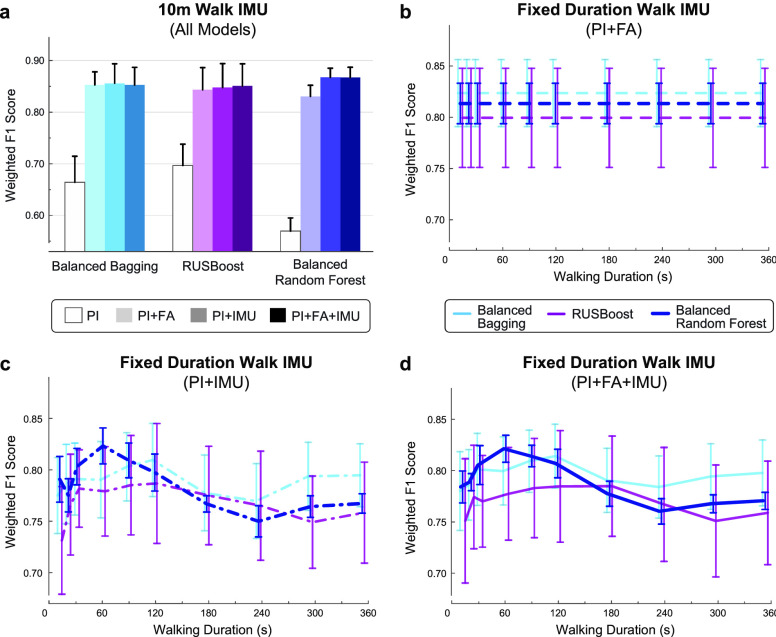
**Algorithm selection for fixed distance and fixed duration models**. Average and SD of weighted F1 score across 100 iterations for three algorithms to predict discharge ambulation outcomes. Pre-optimized performance is shown for different model types trained under the **(a)** fixed distance (10m walk), or **(b-d)** fixed duration (10-360s walk) paradigms, relative to the amount of IMU data used for analysis. Models without IMU data (PI and PI+FA) are unaffected by the amount of IMU walking data. PI models were not considered for the fixed duration analysis given their low performance, shown in **(a)**. The Balanced Random Forest algorithm was selected to compare downstream models for its typically higher performance (e.g., maximum average performance for 10m walk and 60s walk) and lower fluctuation across conditions.

Generally, the Balanced Random Forest classifier demonstrated the highest average weighted F1 score and lower fluctuations in performance (**Fig. 8**). Thus, this algorithm was selected for implementation in the fixed distance and fixed duration analyses.

### G. Model Optimization

Models with the three different combinations of feature inputs (PI+FA, PI+IMU, PI+FA+IMU) were trained and tested using the selected algorithm and leave-one-subject-out cross validation. Prior to testing, we employed feature selection and hyperparameter tuning to optimize each model for maximum performance and reduce risk of overfitting.

**FIGURE 9.**
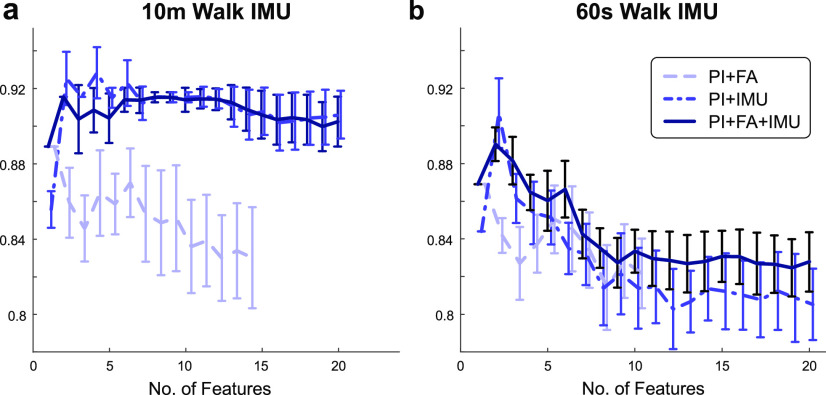
**Feature elimination for fixed distance and fixed duration models**. Average and SD of weighted F1 score across 100 iterations is shown as a function of the number of features, as determined by backward elimination, for **(a)** fixed distance (10m walk), and **(b)** fixed duration (60s walk) paradigms. The subset of features that maximized the weighted F1 score were selected to optimize model training and testing. Performance for the PI+FA model is identical between the fixed distance and fixed duration models since this model is unaffected by the amount of IMU walking data.

In the optimization procedure, we first removed highly correlated features (Pearson’s correlation coefficient >0.9) to avoid multi-collinearity. We then performed recursive feature elimination and cross-validation selection (RFECV function from Scikit-Learn) to identify the most important features for the model and their corresponding importance scores. RFECV was executed iteratively 100 times with different random seeds, resulting in 100 sets of selected features and importance scores. We then summed importance scores for each feature across the 100 RFECV iterations and normalized by the total sum, resulting in importance scores which added to 1 and a cumulative order of importance for the feature set. Finally, we used backward elimination to remove the least important features based on their cumulative order of importance. The mean and standard deviation of weighted F1 scores were calculated using another 100 iterations of the model over different random seeds to capture changes in performance across the number of features used during backward elimination (**Fig. 9**).

Backward elimination indicated that only a subset of features was needed to achieve a maximum average F1 score. For the benchmark model (PI+FA), the only feature needed was 10MWT score at admission. For the fixed distance analysis (with sensor data recorded during a 10-meter walk), the selected features were: standard deviation of acceleration at the pelvis, amount of motion of the stroke-unaffected ankle and the pelvis, and sample entropy of acceleration on the stroke-unaffected ankle (PI+IMU, 4 features); and 10MWT score at admission, amount of motion of the stroke-unaffected ankle, standard deviation of acceleration at the pelvis, skewness of the gyroscope signal on the stroke-affected ankle, sample entropy of acceleration on the stroke-unaffected ankle, amount of motion of the pelvis, sample entropy of the gyroscope signal on the stroke-unaffected ankle, and kurtosis of acceleration on the stroke-affected ankle (PI+FA+IMU, 8 features). For the fixed duration analysis (with sensor data recorded during a 60-second walk), the selected features were: sample entropy of the acceleration signal at the stroke-unaffected ankle and sample entropy of the gyroscope signal on the stroke-affected ankle (PI+IMU, 2 features); and 10MWT score at admission and sample entropy of the gyroscope signal at the stroke-affected ankle (PI+FA+IMU, 2 features).

**FIGURE 10.**
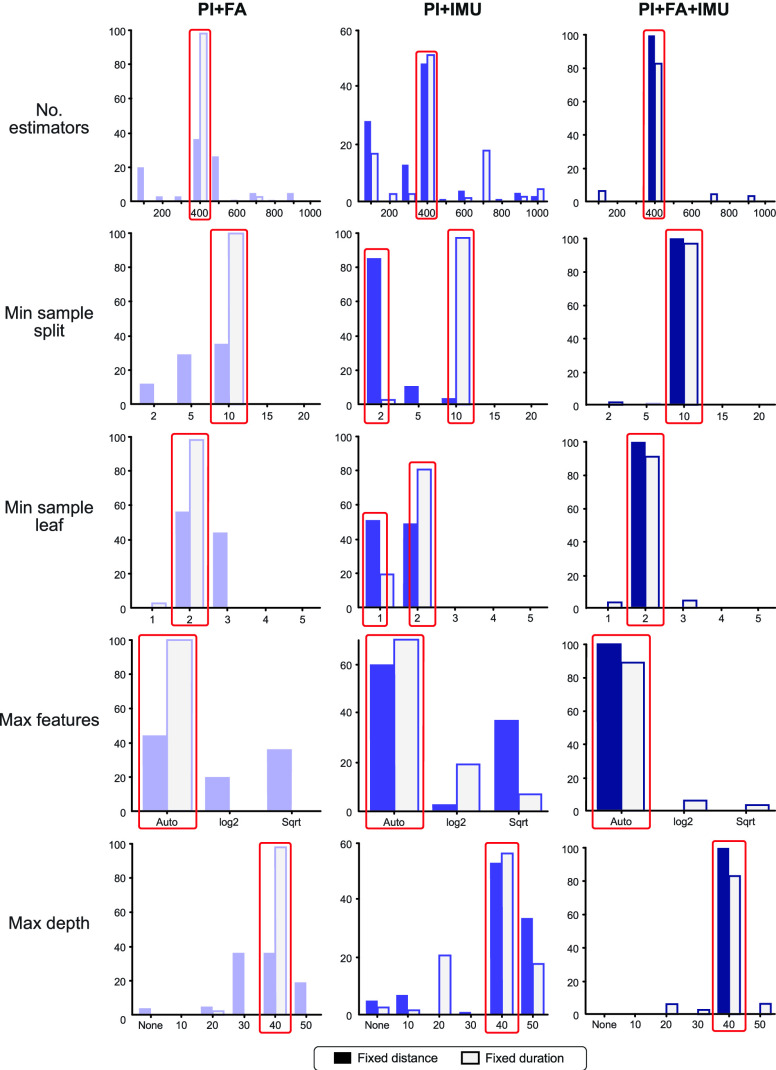
**Hyperparameter selection**. Hyperparameters for the Balanced Random Forest algorithm were tuned using a randomized search cross-validation. Red boxes indicate the values used for each optimized model based on majority-selection from 100 iterations with different random seed states.

Using the selected features, we tuned the hyperparameters of each model based on a randomized cross-validation search (RandomizedSearchCV function from Scikit-Learn, using the default 5 folds). These parameters included the number of estimators, minimum sample split, minimum sample leaf, method for determining the maximum number of features (automatic, log2, or sqrt), and the maximum depth. We executed 100 iterations with different random states and identified the best-performing hyperparameters based on majority vote. The hyperparameters selected for each model using this randomized search approach are shown in **Fig. 10**.

### H. Model Evaluation

Model performance metrics were averaged across test folds (left-out subjects in the cross-validation procedure). Performance was primarily evaluated using the weighted F1 score, which accounts for class imbalances by computing a weighted average of precision and recall based on the number of samples in each class. Secondary model performance metrics included accuracy (proportion of correctly classified samples) and area under the receiver operating characteristic (AUROC). Possible values for these metrics range from 0 to 1, with higher values indicating better model performance.
